# Cytokinin Oxygenase/Dehydrogenase Inhibitors: An Emerging Tool in Stress Biotechnology Employed for Crop Improvement

**DOI:** 10.3389/fgene.2022.877510

**Published:** 2022-03-24

**Authors:** Kavita Arora, Sangeeta Sen

**Affiliations:** ^1^ Department of Botany, National P.G. College, Lucknow, India; ^2^ Bangalore, India

**Keywords:** cytokinins, cytokinin oxygenase/dehydrogenase, inhibitors, stress tolerance, overexpression

## Abstract

In order to meet the global challenges of food security, one of the foremost solutions lies in enhancing the crop productivity. This can be attained by considering key plant hormones such as cytokinins as agrochemicals as cytokinins in particular are known to control the essential processes of the plants. Even though, it has already been established since 1980s that the enzyme, cytokinin oxidase/dehydrogenase (CKO/CKX) deactivates cytokinins; the potential applications of manipulating these enzymes have mostly been speculated to have a high potential in the biotechnology industry and spreads to agriculture, horticulture and agroforestry. The enzyme is critical in maintaining a balanced level of cytokinins in plants. However, it is yet to be fully established that inhibiting this enzyme can be the constant source of improvement in the productivity of plants, even though success has been obtained in some economically important plant species. Furthermore, the impact efficiency of this enzyme may vary from plant to plant, which needs to be evaluated employing tissue culture and other extrinsic applications. This review intends to cover the relevant studies addressing any biological activity of this enzyme in the current context and any associated biotechnological applications specific to enhanced grain yield, abiotic stress tolerance, delayed senescence and *in vitro* organogenesis among various plants and not only cereals. Moreover, our study will identify the present gaps in research with respect to many important food crops, which will be useful for researchers who are actively involved in providing a foundation for a variety of genetically improved plants achieved through this manner. In addition to this, other ways of engineering the amount of cytokinin levels appropriate for signaling also needs to be analyzed in order to extend the benefits of cytokinin biology to other crops too. The application of these inhibitors can be considered among the best alternates as well as addition to genetically modified plants for overcoming the gaps in crop demand.

## Introduction

The exponential rise in human population over the last few decades has forced many ultimate challenges at the basic level in terms of “food, feed, and bioenergy” (Gupta et al., 2021; Nisler et al., 2021), especially for the developing countries, such as India. Moreover, constant human interference has led to environmental imbalance causing poor crop yield. Along with this, various types of abiotic stresses such as drought, salinity, etc., have marred the agricultural production (Aremu et al., 2015). All this has led to scarcity of agricultural land, leaving almost no scope for its expansion to keep pace with the burst to meet the population needs. In order to maintain a sustainable balance between the supply chain of food and demand, it has been strongly realized by the scientists that the solution to this problem lies in focusing on developing ways of enhancing crop productivity of the “existing” agricultural land (Nisler et al., 2021). There are many facets through which the crop yields can be improved; one of such aspect involves controlling the level of plant growth regulators (PGRs) in the crops. It is well known fact that amongst the common PGRs, cytokinins play an indispensable function in plant growth and morphogenesis (Pavlů et al., 2018; Hai et al., 2020). Extensive research on cytokinins have revealed that appropriate levels of cytokinin are necessary for cytokinin governed essential physiological and regulatory responses in different cell types (Gupta et al., 2021) through the “complex network” of cytokinin signaling (Li et al., 2019). These include, controlling the “cell division” involving the expansion, proliferation and development of foliage, branches, root as well as the reproductive organs through “photomorphogenic cell differentiation” (Chiang et al., 2012; Efroni et al., 2013; Bishopp, et al., 2011); non-initiation of lateral roots (Bielach et al., 2012), prolongs stomatal closure (Pospíšilová et al., 2005) and seed fill (Kieber and Schaller, 2014). It has been realized that most of these morphogenetic responses can be directed towards enhancing crop production. Therefore, cytokinins can be employed as “potential agrochemicals” (Koprna et al., 2016; Nisler et al., 2021) for inducing the physiological advantages that can be achieved through enhancing the levels of cytokinins in the plants. Moreover, it has been reported that the increase in cytokinin levels in a plant can enhance seed/crop yields (Bartrina et al., 2011; Jameson and Song, 2016), increase positivity in tillering, improve setting of flowers and seeds (Koprna et al., 2016), impede senescence of the leaf (Zwack and Rashotte, 2015) and mediate their stress tolerance especially in case of drought (Hai et al., 2020; Devireddy et al., 2021), salinity adaptation (Joshi et al., 2018; Li et al., 2019), etc. This review focuses on the current understanding of cytokinin biology in relation to crop improvement. It has been divided into four further sections, commencing with the ways through which the level of cytokinins can be enhanced in the plants, followed by the understanding of the types of the cytokinin inhibitors, their mode of action, then summarizing the various biotechnological responses, especially related to various forms of stress.

## Cytokinin Augmentation in Plants

The enhancement of cytokinins in the plants can be achieved through two possible ways, either by the addition of cytokinins that are natural or synthetic in nature or by restricting the cytokinin inhibitors. Strong natural cytokinins such as zeatin can only be applied to the plant as a “single dose at one time point”, which typically gets diluted after some days (Nisler et al., 2021). The positive impact is visible, however, as a short-term effect rather than a long term one and causes variations that are unreproducible and are therefore, unacceptable from the commercial point of view (Koprna et al., 2016). In contrast, synthetic ones such as thidiazuron (TDZ), N-(2-Chloro-4-pyridyl)-N′-phenylurea (CPPU), etc., are ineffective in their signaling aspects and may induce undesirable side effects.

Besides these, another way to increase the cytokinin levels can be through inhibiting the action of cytokinin regulation. Physiologically within the plants, the levels of cytokinins are controlled through the balance of four enzymes; out of which isopentenyl transferase (IPT), which employs the mevalonate as well as methylerythritol phosphate pathway (Wang et al., 2014), is primarily responsible for the cytokinin metabolism in nature (Jameson and Song, 2016), while deactivation of cytokinin is the sole responsibility of the enzyme called cytokinin oxidase/dehydrogenase, CKO/CKX (Chatfield and Armstrong, 1986; Jiang et al., 2016). As the part of the mechanism of action, CKO/CKX enzyme irreversibly inactivates the cytokinins through the removal of N^6^-isoprene side chain from the cytokinin molecules (Mok and Mok, 2001). It can also be suggested that the CKX enzyme, being a flavoprotein (Gupta et al., 2021), is also involved in the balance as well as regulation of cytokinins, thereby helps in maintaining cytokinin homeostasis (Thu et al., 2017; Hai et al., 2020). This regulatory function has mostly been reported from major cereals such as *Hordeum vulgare* (Zalewski et al., 2014), *Zea mays* (Brugière et al., 2003), *Oryza sativa* (Ashikari et al., 2005) and *Triticum aestivum* (Song et al., 2012; Zhang et al., 2012; Ogonowska et al., 2019). At the genetic level, the prevalence of *CKX* gene families in plants has varied from species to species (Nisler et al., 2021) with isoforms differing in “spatial and temporal expression patterns and subcellular localization” with some being localized in the apoplast, vacuoles and cytosols (Joshi et al., 2018; Nisler et al., 2021). The number of genes involved in cytokinin inhibition ranges from seven as found in *Arabidopsis thaliana* (Werner et al., 2003) and *Medicago sativa* (Li et al., 2019) to eight in *Fragaria vesca* (Jiang et al., 2016), eleven in *Oryza sativa* (Tsai et al., 2012) and *Triticum aestivum* (Chen et al., 2020), twelve in *Malus domestica* (Tan et al., 2018), thirteen in *Zea mays* (Morris et al., 1999) and 23 in *Brassica napus* (Liu et al., 2013). These genes can be targeted for production of genetically modified plants, which will induce the overexpression of CKX enzyme and can cause drastic changes in the “organ proportions” especially root morphology in barley plants as observed by Mrί;zová et al. (2013). The negative regulation of the cytokinins leads to enhanced crop yield and mediation towards tolerance of abiotic stresses as reported in rice (Yamburenko et al., 2017), *Arabidopsis* (Werner et al., 2003; Prerostova et al., 2018), barley (Pospíšilová et al., 2016; Holubová et al., 2018). Besides the up regulation of this gene, its down regulation or knocking off has also caused increased yield in rice due to the increase in the quantity of reproductive organs (Ashikari et al., 2005) even during salinity stress (Joshi et al., 2018). Apart from the traditional forms of genetic modification such as selective breeding and crossbreeding, genetic engineering and genome editing are some of the mechanisms through which gene manipulation can be done (US Food and Drug, 2022). It was reported that controlling this enzyme can lead to “tailor made” improvements in the productivity of plants (Ashikari et al., 2005). Moreover, newer techniques for genome editing such as CRISPR/Cas9 (clustered regularly interspaced short palindromic repeat) have been recently used for knocking out of *CKX/CKO genes* in barley (Holubová et al., 2018; Gasparis et al., 2019) and rice (Mao et al., 2020; Rong et al., 2021). However, none of the mechanism of action has not been fully understood till now (Joshi et al., 2018), even though success has been obtained in some economically important plant species such as apple (Liao et al., 2017), tobacco (Macková et al., 2013), etc. Furthermore, the effectivity of the impact of this enzyme may vary from plant to plant, which needs to be evaluated employing tissue culture and other extrinsic applications (Gupta et al., 2021).

## Cytokinin Oxygenase/Dehydrogenase Inhibitors: Types and Mode of Action

**FIGURE 1:**
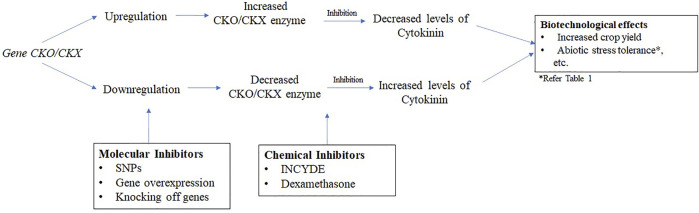
Mechanism of *CKO/CKX* gene control.

The primary approaches to decrease the expression of CKX enzyme can either be through chemical means (Kopecný et al., 2010; Nisler et al., 2021) and molecular approaches (Gouda et al., 2020a; Nguyen et al., 2021). Figure 1 represents a schematic diagram on the mechanism of *CKO/CKX* control. Nisler et al. (2021) points that inhibition of CKX enzyme by chemicals had been reported long time back which is predated even before the engineering of the genetically modified plants. These chemicals are classified as synthetic cytokinins such as TDZ and its variants (Nisler et al., 2016, Nisler, 2018), diphenyl urea (DPU), chloropyridin phenyl urea (CPPU), N-(2-amino-pyridin-4-yl)-N′-phenylurea (APPU) (Kopecný et al., 2010) or new potent inhibitors derived from CPPU, DPU, and DCPU (Nisler et al., 2021). The findings from Nisler et al. (2016) showed a 15-times decrease in half-maximal inhibitory concentration (IC50) with TDZ for *AtCKX2* in *Arabidopsis* and *ZmCKX1* and *ZmCKX4a* in *Zea mays*. Along with this, derivatives of 2-X-6-anilinopurine along with 2-chloro-6-(3-methoxy- phenyl) aminopurine (INCYDE) have also been found to be effective inhibitors of CKX enzyme in *Arabidopsis* (Zatloukal et al., 2008; Prerostova et al., 2020) and tomato (Aremu et al., 2014), respectively. The antioxidant defense mechanism and efficiency of photosynthesis got elevated by the use of these potent compounds (Aremu et al., 2014). The potency of inhibition was found to be higher in the variant of DPU in comparison to DCPPU and the inhibition occurred at the concentration of 10^−8^ M (Nisler et al., 2021). Similarly, APPU was found to be a better inhibitor as compared to CPPU, TDZ and their derivatives (Kopecný et al., 2010). Moreover, the chemical use of CKX enzyme inhibitors was found to be more advantageous than the application of cytokinin exogenously as a moderate level but “long-term” enhancement in the endogenous levels of cytokinins was observed. Among the molecular approaches, heterogenous nuclear RNA (hRNA-CX3 and -CX5) were used to suppress expression of CKX enzyme in rice (Yeh et al., 2015). An increase in growth, chlorophyll content and grain yield were observed in this case. Recently, one of the molecular approaches applied specific missense single nucleotide polymorphisms (SNPs), namely SNP42, SNP43, SNP44, and SNP46 to reduce the expression of CKX enzyme in rice that led to increase in grain numbers (Gouda et al., 2020a), while another nine SNPs from five genes were demarcated in soybean for enhanced seed yield (Nguyen et al., 2021). In a new approach, computational means has also been followed to study the “structure, function and interaction” of the CKX enzyme from rice plants for the first time (Gouda et al., 2020b). A hypothetical 3-D structure of this enzyme was predicted, which showed the presence of 24 α helix and 13 β strands. This can be extremely useful in understanding the cause of enhanced yield in these plants.

## Biotechnological Responses

The decrease in CKX enzyme using various form of inhibitors has manifested a series part of the biotechnological application response or effects. Table 1 summarizes the various studies conducted on the understanding the influence of CKX enzyme inhibitors over abiotic stress tolerance. One of the most common manifestations observed in the genetically modified plant includes the reduction of abiotic stresses and adaptations to drought in *Arabidopsis* (Prerostova et al., 2018), barley (Pospíšilová et al., 2016; Ramireddy et al., 2018), tobacco (Werner et al., 2010; Macková et al., 2013; Lubovská et al., 2014) and apple (Liao et al., 2017); heat tolerance in *Arabidopsis* (Prerostova et al., 2020); cold as well as salinity tolerance in tomato (Aremu et al., 2014), in alfalfa (Li et al., 2019), in *Arabidopsis* (Nisler et al., 2021), etc. Moreover, tolerance towards stresses from heavy metals such as cadmium also can be observed as a result (Gemrotová et al., 2013). Most importantly, there is an increase of antioxidant enzymes (Devireddy et al., 2021). Other outcomes include inducing shoot regeneration, roots and morphogenesis in Chinese water chestnut (Wang et al., 2015), *in vitro* responses such as organogenesis (Aremu et al., 2015; Werbrouck, 2016; Chen and Wei, 2018; Mazri et al., 2018), callus culture bioassays (Kopecný et al., 2010), delayed senescence (Nisler et al., 2016; Prernostava et al., 2018) and as basic as increasing yield of the cereal crops (Ashikari et al., 2005; Nisler et al., 2021). Moreover, 44% increased zinc levels were present in the seeds of the transgenic barley plant along with drought tolerance. It was construed that the overexpression of CKX enzyme made the plant more nutrient efficient (Ramireddy et al., 2018). In contrast, Gasparis et al. (2019) reported that knocking out the *CKX* genes may not enhance the grain yield in barley.

**TABLE 1:** Biotechnological responses of plants targeted with CKX inhibitors through chemical and molecular approaches.

Sl	Plant name	Chemical/Molecular approaches	CKO/CKX family member or gene targeted	Biotechnological applications/ response/ effects	Reference
1	Soybean	Molecular- SNPs	*GmCKX GFMs,*	Increased yield and proposed abiotic stress resistance	Nguyen et al. (2021)
2	Maize, *Arabidopsis*, Spring barley, Winter wheat, winter oilseed rape	Chemical- new inhibitors derived from DPU	*AtCKX 2, Zm CKX1, ZMCKX4q* and *ZmCKX8*	Stress resistance and increased seed yield in *Arabidopsis*	Nisler et al. (2021)
3	*Arabidopsis*	Chemical- INCYDE	Not mentioned	Heat tolerance	Prerostova et al. (2020)
4	*Arabidopsis*	Molecular- Overexpression of genes in the genetically modified plant	Introduced *MsCKX from Alfalfa*	Salt tolerance	Li et al. (2019)
5	*Arabidopsis*	Chemical- Dexamethasone	*AtCKX1*	Drought tolerance	Prerostova et al. (2018)
6	Barley	Molecular- Overexpression of genes in genetically modified plant	Introduced *AtCKX1*	Drought tolerance	Ramireddy et al. (2018)
7	Rice	Molecular – Knocking off in genetically modified plant	*OsCK2*	Yield increase and salinity tolerance	Joshi et al. (2018)
8	Apple	Molecular- Overexpression of genes in genetically modified plant	*MdCKX4a*	Drought tolerance	Liao et al. (2017)
9	Tomato	Chemical: 2-chloro-6-(3-methoxy- phenyl) aminopurine (INCYDE)	Not mentioned	Salt tolerance, vegetative and reproductive growth	Aremu et al. (2014)
10	Medicinal plants- *Bulbinea* and Curly dock	Chemical- INCYDE	Not mentioned	Adaptation towards cadmium stress	Gemrotová et al. (2013)
11	Tobacco	Molecular- Genetically modified plant	Introduced *AtCKX1*	Drought and heat tolerance	Macková et al. (2013)

## Conclusion, Perspectives and Future Scope of Research

The application of inhibitors of CKX enzyme as a successful and capable tool for tolerance of abiotic stresses is evident from this study, which has a great potential for crop improvement in a variety of crops, including cereals. The present study reviews relevant research pertaining to the biological activity of the CKX enzyme in the context of adapting towards abiotic stresses along with improved grain yield. This can also be extended as the source of providing benefits to various crops through cytokinin biology. Other biotechnological responses of this enzyme also include delayed senescence and inducing organogenesis through tissue culture. In addition to this, other ways of manipulating the level of cytokinin suitable for signaling was also explained and the present gaps in this research area has been identified from this study.

The comparison of CKX enzyme inhibitors reveals that the use of chemicals is more popular over the molecular approaches. Therefore, it is anticipated that these chemicals can work as an alternate to genetically modified crops (Nisler et al., 2021). This will be extremely advantageous for mankind as any legal hassles towards acceptance of genetically modified organisms (GMOs) can be easily avoided using this approach, implying a wider reach among many varieties of plants across countries. However, an appropriate dosage level as well as the “cost effectiveness” of these chemicals is yet to be assessed at a commercial level, thereby warranting immediate attention from the researchers in this field. Recent studies demonstrate the emergence of successful genetic approaches (Wang et al., 2020; Nguyen et al., 2021; Nisler et al., 2021; and many others), emphasizing that modulating CKX enzymes can open up multiple paths for developing “tailor made” stress resistant and nutrition rich crops which will be useful in the long-term breeding programs (Ramireddy et al., 2018). These will be developed as a means of sustainable agriculture through unravelling the signaling network of the cytokinins (Pavlů et al., 2018). From this review, it was also realized that both up- and downregulation of the *CKX* gene can be instrumental in improving the economic needs, even though it seems to vary from plant to plant and even within a plant species. This ambiguity opens up a wide scope for further molecular research. In future, CKX inhibitors can be treated as part of plant defense regulators and studies can focus on comprehending the molecular mechanism of the interaction of CKX enzyme with other plant defense regulators such as jasmonic acid, salicylic acid, ethylene, abscisic acid (ABA) and others in order to develop a better understanding towards abiotic stresses.
